# Supplementary figures and images for: Selection and Validation of Reference Genes for Gene Expression Studies Using Quantitative Real-Time PCR in Prunus Necrotic Ringspot Virus-Infected Cucumis sativus
Source: Viruses. 2022 Jun 10;14(6):1269. doi: 10.3390/v14061269 (PMC9227502; doi:10.3390/v14061269)

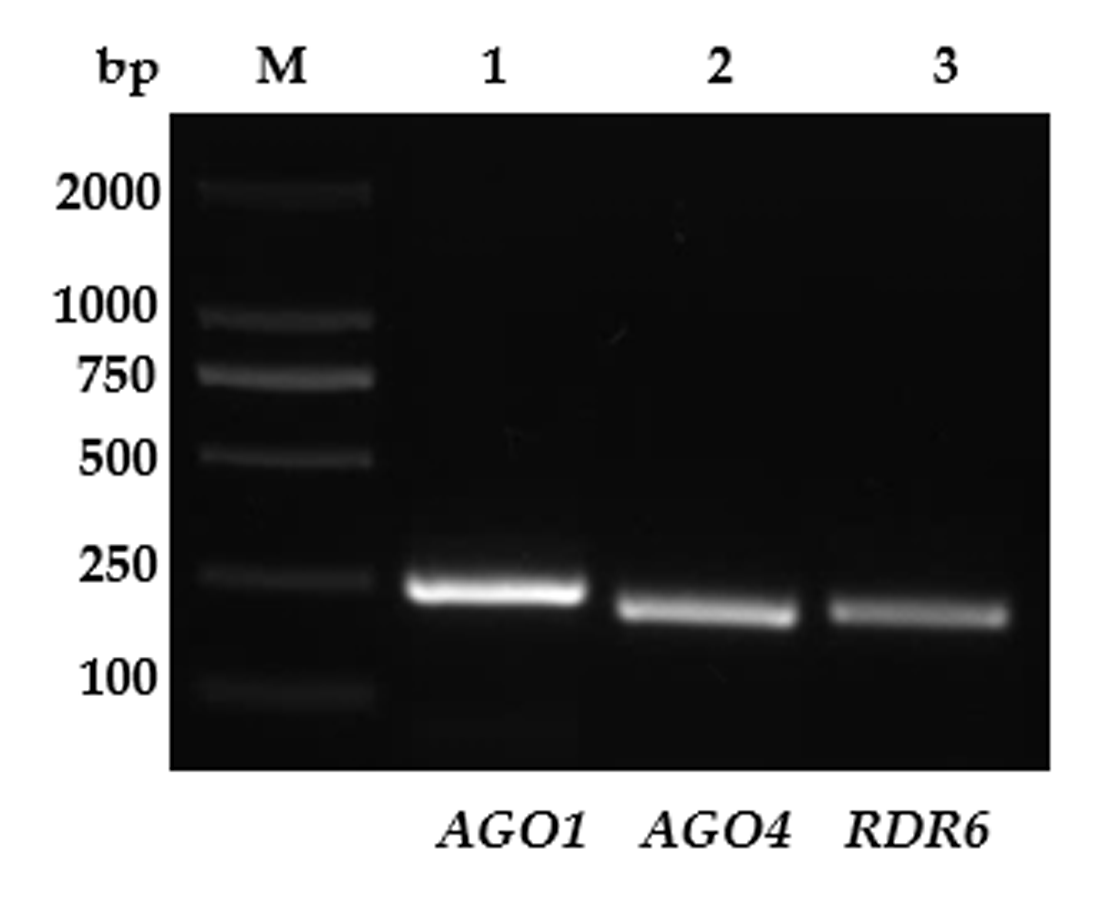

Supplement: Supplementary file 1 [file viruses-14-01269-s001.zip › Figure S1.tif]

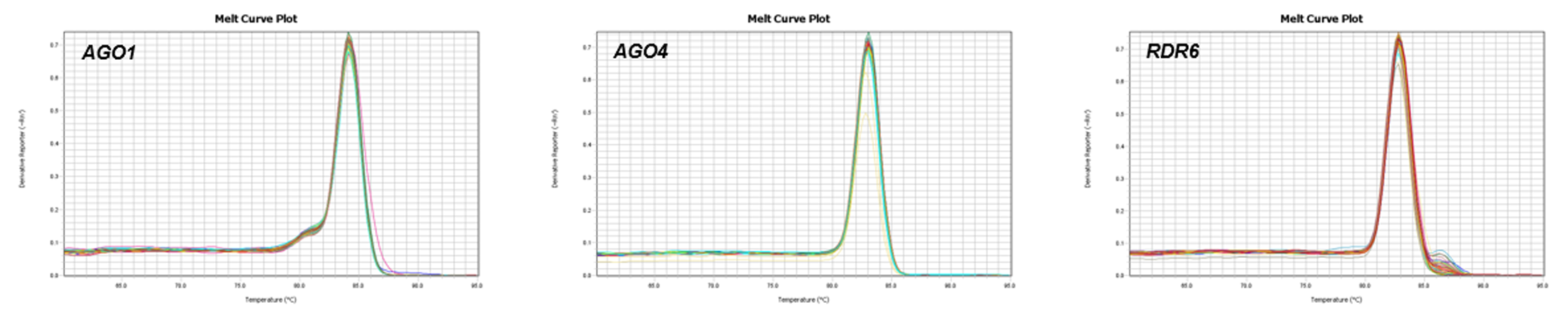

Supplement: Supplementary file 1 [file viruses-14-01269-s001.zip › Figure S2.tif]
